# Political struggles for a universal health system in Brazil: successes and limits in the reduction of inequalities

**DOI:** 10.1186/s12992-019-0523-5

**Published:** 2019-11-28

**Authors:** Cristiani Vieira Machado, Gulnar Azevedo e Silva

**Affiliations:** 1 Sergio Arouca National School of Public Health, Oswaldo Cruz Foundation, Rio de Janeiro, Brazil; 2 Social Medicine Institute, State University of Rio de Janeiro, Rio de Janeiro, Brazil

**Keywords:** Health policy, Health inequalities, Health reform, Unified health system, Brazil

## Abstract

**Background:**

Brazil is a populous high/middle-income country, characterized by deep economic and social inequalities. Like most other Latin American nations, Brazil constructed a health system that included, on the one hand, public health programs and, on the other, social insurance healthcare for those working in the formal sector. This study analyzes the political struggles surrounding the implementation of a universal health system from the mid-1980s to the present, and their effects on selected health indicators, focusing on the relevant international and national contexts, political agendas, government orientations and actors.

**Main text:**

In the 1980s, against the backdrop of economic crisis and democratization, Brazil’s health reform movement proposed a Unified Health System (SUS), which was incorporated into the 1988 Constitution. The combination of a democratic system with opportunities for interaction between various developmental and social agendas and actors has played a key role in shaping health policy since then. However, the expansion of public services has been hampered by insufficient public funding and by the strengthening of the private sector, subsidized by the state. Private enterprises have expanded their markets and political influence, in a process that has accelerated in recent years. Despite these obstacles, SUS has produced significant health-status improvements and some (although incomplete) reductions in Brazil’s vast health inequalities.

**Conclusions:**

We find that a combination of long-term structural and contingent factors, international agendas and interests, as well as domestic political struggles, explains the advances and obstacles to building a universal system in an economically important yet unequal peripheral country. Further consolidation of SUS and reduction of health inequalities hinge on the uncertain prospects for democracy and national development, on enlarging the political coalition to support a public and universal health system, and on strengthening the state’s ability to regulate the private sector.

## Background

Brazil is a territorially vast, populous, high/medium-income federal republic in the periphery of global capitalism, widely recognized as one of the world’s most unequal countries. Its economic and social inequalities are evident in epidemiological data, access to and outcomes from the health system, across regions and demographic groups [1].

Like other Latin American countries, Brazil’s health system during most of the twentieth century was characterized by public health programs that focused on the control of specific infectious diseases, combined with medical assistance services intended for urban workers in the formal sector, according to a logic of social insurance. Between the 1930s and 1980s, the country underwent a process of state-induced industrialization that emphasized import substitution, and an accompanying process of rapid urbanization. Significant demographic changes occurred, due to declining mortality and fertility and increasing life expectancy. Health indices showed an epidemiological transition characterized by a rise in cardiovascular illnesses, cancer diseases, and external causes (violence and accidents), accompanied by the persistence of older infectious diseases (tuberculosis, Hansen’s disease) and the emergence of others [2].

The expansion of pension systems and access to public health services took place mainly under authoritarian governments, with limited social participation. From the 1960s, there were increasing state incentives for the private sector, with a strengthening of the corporate health-care industry over the ensuing decades. This occurred both through the contracting of private health services—mainly hospitals—by social insurance institutions and through fiscal incentives for businesses to offer private health plans to their employees [3, 4].

Amidst economic crisis and democratization in the 1980s, Brazil underwent a process of healthcare reform that culminated in the recognition of health care as a right of citizenship and the creation of the public, universal Unified Health System (SUS) enshrined in the Constitution of 1988. This system was to be tax-funded, comprehensive and universally accessible to all Brazilians, free of charge, regardless of their economic or social status. Brazil was the only Latin American country to propose a universalistic health reform in the 1980s, but implementation proved difficult in the following decades. What political factors led to the introduction of a universal health system in Brazil, in contrast to the predominant neoliberal international trends in healthcare reform elsewhere in Latin America? In the face of a strong private sector, which were the political forces that supported or resisted making SUS a truly universal system over the following decades? In a context of deep social inequalities, has SUS served to reduce health inequalities?

This paper analyzes the political struggles over the implementation of a universal health system since the mid-1980s and their effects on selected health indicators in Brazil, during three decades of democratic rule. While recognizing the importance of the structural determinants of health policies, it focuses on the political factors (actors, agendas, power relations, interests) that enable or pose limits to ensuring health as a right of citizenship in a populous, middle-income and unequal Latin American country.

The policy analysis comprises three moments—democratic transition and healthcare reform (1985–1989); the political struggle over SUS in the democratic period (1990–2015); and political crisis, democratic instability, and threats to SUS (2016–2018). Finally, we present some selected health indicators and discuss the achievements and limits in building a universal health system in Brazil.

### Democratic transition and healthcare reform (1985–1989)

Starting in the late 1970s and early 1980s, the international debate surrounding the crisis of the nation-state and the neoliberal agenda began to reverberate in Latin America. Some countries, like Mexico and Chile, were influenced by early neoliberal economic reforms, also with effects on their health policies [5].

While other countries in the region were moving toward a neoliberal model, Brazil took a somewhat different path. During these years, it experienced a serious economic crisis, criticisms of the model of import substitution industrialization (ISI), and a movement toward democratization after nearly two decades of military dictatorship. Brazil also experienced intense social mobilization in favor of progressive reforms.

It was in this context that the movement for healthcare reform emerged, seeking to transform a health system that was segmented, fragmented, inefficient, and oriented toward privileging the private sector while excluding most of the population. The healthcare movement brought together various groups seeking to construct an agenda for reform of this sector. Key groups involved included academics at university departments of preventive medicine or public health, administrators, and experts from the federal Ministry of Health and from the health bodies connected to the Ministry of Social Security, and health professionals, among others. These years also witnessed the formation of the Brazilian Center for Health Studies (CEBES), the Brazilian Postgraduate Association in Collective Health (ABRASCO), and national councils of state and municipal secretaries of health. These healthcare professionals joined with other social movements, including community-based movements associated with the Catholic Church and progressive politicians to construct a reform agenda [6, 7].

Successful experiences with reorganizing health care systems at the local level, along with the presence of progressive public health officials in national posts, set the stage for gradual transformations in healthcare institutions, even as a national reform agenda was created, based on the recognition of health as a right of citizenship. During a 1979 symposium in the Chamber of Deputies (the lower house of Congress), CEBES presented a paper focusing on the relationship between democracy and health [8].

After nearly two decades, gubernatorial elections were held in 1982, and elections for mayors of state capitals and cities designated as “national security” zones in 1985. Also in 1985, Congress indirectly elected the first civilian president since 1964. With the death of the President-elect before his inauguration, the Vice President-elect took office and assembled a broad coalition government.

In 1986 the Eighth National Health Conference brought together over 4000 participants from across the country—academics, administrators, health professionals, social movements, and ordinary citizens—who advocated for the strengthening of the public system and for the designation of health as a right. The conference led to the formation of the National Committee for Health Care Reform, which elaborated a reform proposal that was presented to the 1987–1988 National Constitutional Convention. Brazil already had an important private health sector, with private hospitals contracted by public social security institutions, as well as a growing sector of private health insurance plans. These groups pressured legislators to avoid proposals that could result in a radical shift toward state control and the imposition of constraints on the private sector [9].

The 1988 Constitution recognized health as a universal right that the state was required to provide, guaranteed by broad social and economic policies. It also institutionalized the concept of “social security”—comprising health, pensions, and social assistance—and the Unified Health System (SUS), a public, universal system intended to ensure comprehensive health care for the population. The Constitution affirmed the complementarity of the private sector, with priority for philanthropic and non-profit institutions. It also stated that health care would be “open for private investment,” thus retaining openings for expansion of the private system, even as it failed to address important questions regarding public financing of healthcare.

The global transformations of the 1970s and 1980s affected Brazil, with implications both economic (economic crisis, the exhaustion of ISI) and political (democratization). However, the national context better explains social changes, including in health care. The international agenda of neoliberal reforms did not have the same impact on Brazil in the 1980s as elsewhere in Latin America. The temporal sequence of two processes—democratization and economic liberalization—and the promulgation of a comprehensive Constitution served to shield Brazilian social policies from the neoliberal reforms: they would begin later, in a less aggressive and more pragmatic form [10]. The return to democracy in the 1980s created an atmosphere conducive to mobilization for universal healthcare reform, with the support of state and local governments and legislators. The inauguration of a civilian president and the calling of a National Constitutional Assembly, amidst a climate of intense debates over the future of the country, played a significant role in enabling the creation of SUS, a system inspired by the experiences of other countries, like the United Kingdom’s National Health Service (NHS) and Italy’s healthcare reform.

On the other hand, several key political actors were not on board with the SUS agenda. For example, the businesspeople who controlled the private sector sought to protect their market share. The labor movement expressed inconsistent positions regarding the conflict between weakening workers’ access to healthcare and universalizing the system; they also lobbied employers to provide private plans. For their part, although medical doctors rarely oppose SUS directly, and the organizations representing them espoused a range of positions, their responses to its agenda were motivated primarily by their collective professional interests.

Over the following decades, these conflicts of interests and projects would surface forcefully. Two aspects—restrictions on public financing, and the nature of public–private healthcare relations—emerged with the difficulties in constructing a universal public system capable of helping to overcome the fragmentation of the system and reduce healthcare inequalities.

### Political struggle over SUS in the democratic period (1990–2015)

Like other Latin American countries in the 1990s Brazil adopted neoliberal reforms that involved economic opening, the reining-in of public spending, reduction of the size of the state apparatus, and privatizations of state enterprises. This agenda was launched by the liberal government of Fernando Collor (1990–1992). It slowed down under the transitional government of Itamar Franco (1992–1994), who took office after Collor resigned amidst a process of impeachment; and was taken up again, with new contours, during the two terms of Fernando Henrique Cardoso (1995–1998; 1999–2002).

Also influential in Latin America were healthcare reform proposals promoted by international agencies, among them the World Bank [11]. Their recommendations included the separation of funding from the provision of services; the establishment of cost-effective basic service packages; and focusing state action on the poorest citizens.

In Brazil, the struggle for the creation of SUS, combined with the constitutional guarantee of health as a right, prevented the direct adoption of specific World Bank health proposals. However, the subsequent trajectory of health policy made clear the inherent tensions between a free, universal healthcare system like SUS and state-driven market reforms. The former was promoted mainly by the public health system and civil society actors; the latter was defended by government economic authorities (Ministers of Finance and Planning and Budget) and by the owners of private health enterprises, wishing to expand their share of the market.

Among those advocating the expansion of the public system, a coalition coalesced around the development of a legal-institutional framework, which, in addition to the broad principles of universality and comprehensive care, envisioned federative cooperation and social participation in policy-making. Intergovernmental health commissions were formed at the national and state levels to negotiate the decentralization of power and allocation of resources to state and local governments. In addition, Brazil created health councils at the federal, state (26 states plus the Federal District), and local (over 5000 municipalities) levels that included administrators, providers, professionals, and users of the system. Decentralization, the health councils, and the expansion of public services increased the number of actors with a stake in defending SUS—administrators and experts from all three levels of government, social movements, and users.

Also important were the social groups, at times working with international actors, that came together to propose specific policies based on the SUS principles of universal coverage and comprehensive care. For example, mental healthcare reformers emphasized the implementation of innovative services and programs, such as the expansion of Psychosocial Community Centers and the Return Home program to deinstitutionalize long-stay patients. Their attempts at asylum closure clashed with the interests of private providers [12]. Another example was the policy offering comprehensive care to people with HIV/AIDS, with a focus on prevention and providing new treatments that were emerging in the 1990s. Collaboration involving civil society, experts, healthcare professionals, and the judicial system was central to the development of a policy that guaranteed access to treatment. By ensuring public access to expensive drugs protected by patents, Brazil’s HIV/AIDS policy put the country in the spotlight of global debates and negotiations on intellectual property and the right to health in developing countries [13]. A third example concerned policies related to the control of tobacco, in which Brazil took a pioneering role through a series of initiatives in the 1990s to regulate advertising and use. These initiatives placed Brazil at the forefront of international discussions surrounding the formation of the UN Framework Convention on Tobacco Control, established in 2003 [14]. Yet another innovative policy that gained international recognition was the Family Health Program initiated in 1994, noteworthy for its emphasis on primary care and the way it brought together a range of actors in support of expanding access and changes to the healthcare model. The program was designed in accordance with the core SUS principles of universality and comprehensiveness, further expanded with incremental innovations under various governments. Ultimately, it came to cover much of the country over the next two decades, gaining international recognition for its comprehensiveness and cost-effectiveness [15].

However, implementation of a universal system in Brazil was rendered difficult due to the market-oriented reform agenda adopted by the federal government and various states, which imposed restrictions on public funding and the expansion in the healthcare professionals and supplies needed for a public, universal healthcare system. The struggle to stabilize and increase public funding mobilized actors from across the healthcare system—federal health ministers, state and health secretaries, healthcare professionals, groups of users—throughout the decade. Attempts to create a specific tax on financial transactions in 1996 and a constitutional amendment (approved only in 2000) helped to stabilize the system, but were not enough to guarantee a meaningful increase in state support for healthcare.

Thus, from the beginning, the expansion of SUS services and coverage took place under adverse financial conditions. The system remained dependent on contracting private services, which continued to play an important role in hospital, diagnostic, and therapeutic services. New public–private linkages appeared, such as outsourcing and the contracting of “social organizations” to provide certain services within public facilities—first in hospitals, then in specialized clinics, and eventually even in primary healthcare services. The boundaries between public and private spheres became less clear, favoring the transfer of resources from the state to the private services and organizations. The private insurance sector continued to grow, lobbying governments for its own interests. In keeping with other attempts at regulation by the Ministry of Health, in 1998 Congress passed a Health Insurance Plan Law, and in 2000 a national agency was created to regulate private health plans [16].

In the 2000s, several important countries in Latin America experienced a political “left turn,” [17], stemming, in part, from widespread dissatisfaction with the effects of the neoliberal reforms of the preceding decades. Progressive governments implemented policies expanding the state’s role in the economic and social realms, achieving reductions in inequality. By the middle of the decade, the commodities boom had come to play an important role in contributing to such policies, but they were also a result of the political orientation of Latin American governments.

The “left turn” came to Brazil in 2002, with the election to the presidency of Luis Inácio Lula da Silva, a former metalworkers’ union leader and founder of the Workers’ Party (PT). During Lula’s two terms (2003–2006; 2007–2010), economic tensions persisted between the promotion of austerity and attempts to resume a developmentalist agenda, especially during the second term. These tensions were exacerbated during the government of his successor, also from the PT, Brazil’s first female president, Dilma Rousseff (2011–2014; 2015–May 2016). She had to govern in a less favorable economic context, with the end of the commodities boom, and in the face of formidable political opposition, which culminated in her impeachment and removal from office in 2016, charged with utilizing illegal budgetary measures.

The labor policy of the PT governments focused on attempts to formalize labor relations and increase the real value of the minimum wage. Changes in foreign policy prioritized creating a new international geopolitical alignment, with an emphasis on South–South cooperation with South American and African countries, also in healthcare.

In social policy, the Lula and Dilma presidencies expanded conditional cash-transfer programs, in keeping with the poverty-fighting agenda across Latin America. They also worked to expand rights for socially vulnerable groups (women, Afro-Brazilians, LGBTIQ+ people, indigenous people, and rural communities descended from escaped enslaved people). Their education policy feature the expansion of access to federal and private universities, with publicly-funded scholarships. The link between economic and social policy stimulated a certain dynamization of the internal market and helped reduce poverty and income inequalities, although they still remained high. The commodities boom during Lula’s second term enabled the expansion of social investment and reduction in inequality to occur with only limited resistance. Even amidst the global economic recession of 2009, social spending in Brazil exhibited counter-cyclical behavior.

Especially during Lula’s second term, the focus on “social-developmentalism” manifested itself in healthcare policy, through debates on the relation between healthcare and development, and initiatives to incentivize the domestic production of medication and medical supplies, both outlined in SUS priorities. Under the Lula and Dilma governments, new health programs were created, along with incremental policy innovations that enabled the expansion of access in areas like oral health, urgent care, access to medication, without particularly radical changes. Noteworthy was the progressive increase of primary-care coverage, through the 1994 Family Health Strategy, along with the incorporation of other healthcare professionals to the network of primary-care teams of doctors, nursing professionals, and community health workers [18].

The coalition of actors defending SUS remained the same—administrators from all three levels of government, experts, and professionals, allied with groups of users and members of the judicial system. A caveat is in order concerning doctors, who usually practice in both the public and private systems. Throughout the period studied here, doctors joined together to defend their collective interests—career, autonomy, remuneration—whether engaged in dialogue with public authorities or in their negotiations with private providers and healthcare corporations. Under Dilma’s government, the “More Doctors” program—aimed at hiring doctors to practice in poorly-served regions and communities, creating new degree programs in Medicine, and instituting curricular changes—unleashed conflicts with the medical profession. The principal reason was the contracting of foreign doctors without requiring that they revalidate their diplomas with the Federal Council of Medicine: this was perceived as showing lack of respect for the principle of professional self-regulation, and as a threat to the labor market for Brazilian doctors. The contracting of Cuban doctors, via an accord mediated by the Pan-American Health Organization [19], encountered particularly strong opposition. Also criticized was the hasty creation of degree programs without adequate quality control.

Regarding public funding, there was significant mobilization throughout this period. During Dilma’s government, the “Health Plus 10” movement sought to ensure that 10% of gross federal tax revenues would be reserved for healthcare. However, the legislative measures for funding healthcare were inadequate, and the difficulties with funding the public system remained.

The private sector continued to expand dynamically, diversifying its economic and political strategies. The process of financialization accelerated, via business mergers, new financial market strategies, and the growing penetration by foreign corporations of Brazilian markets [20], despite constitutional restrictions on foreign capital in this field. In the political realm, healthcare corporations reorganized themselves, with new representative organizations, heightened lobbying of Congress, and financial contributions to executive and legislative electoral campaigns.

The state agency created in 2000 to regulate private health plans has focused on regulating contracts, systematizing information, and organizing the market—but never on restricting the growth of the private sector. To the contrary, it has frequently had directors from the very sector they are supposed to regulate.

In December 2014, two months after elections that brought a second term for Dilma Rousseff, the President issued an executive order authorizing the entry of foreign capital in the field of healthcare, including service provision, which was prohibited by the 1988 Constitution. Despite protests from various pro-SUS organizations which held that this was unconstitutional, in 2015 Congress passed the executive order into law.

This legal change led to an expansion in the role of foreign healthcare corporations in Brazil and their subsequent alliance with the large philanthropic hospitals and agencies seeking to regulate private healthcare plans. This constituted yet another of the growing concessions the President made to the corporate sector, in the face of congressional opposition due to decreasing economic growth and overall lack of governability.

In summary, various policy agendas and actors influenced Brazil’s health policy between 1990 and 2015. The political coalition in defense of SUS involved mainly sectoral actors—health authorities, officials, professionals and academics—that became more diversified as the public services were expanded; new actors also became relevant, with some public prosecutors and new social movements. On the other hand, each Presidential coalition in this period involved alliances with conservative groups, and the economic authorities favored market-oriented reforms that were detrimental to SUS expansion and funding. Health enterprises became more dynamic and international, and intensified political lobbying. Finally, unions and doctors’ organizations tended to focus on their specific group interests.

### Political crisis, democratic instability, and threats to SUS (2016–2018)

Throughout 2015, the political crisis in Brazil intensified, aggravated by a national economic crisis. The Vice-President, Michel Temer, engineered the impeachment of President Rousseff and launched a neoliberal reform package to placate the “markets.” With the support of corporate elites, politicians, and the media, the process culminated in Rousseff’s suspension from the presidency in May 2016, following controversial accusations of illegal budgetary measures—and her definitive removal from office by the Senate in August 2016.

A new era began for Brazil, one in which new political actors took center stage, with threats to social policy, the healthcare system, and democracy itself. The impeachment has been called a “parliamentary coup,” supported by the judiciary and the media and aimed at removing the PT from power, after its four consecutive victories in presidential elections [21]. The imprisonment of Lula da Silva in 2018 on allegations of corruption was supported by evidence that was shaky at best, but it succeeded in impeding him from running for President, and in so doing offered more support for the argument that Brazilian democracy is under attack.

Further indications of the fragility of Brazil’s democratic pact came with the rapid adoption of a reform agenda that voters had not approved when they voted for Dilma in 2014. Soon after assuming the Presidency, Temer began to implement neoliberal measures, with an emphasis on economic austerity, reduction of the size of the state apparatus, changes to the then-current social pact, and market incentives. Within the executive branch, he promoted a drastic reduction in the number of cabinet ministries by merging some key ministries and abolishing others. Working with Brazil’s most conservative Congress in half a century and supported by the corporate elite, Temer signed a labor reform bill which loosened rules regulating labor and restricted worker rights. His government also gained approval of a constitutional amendment that froze social spending for 20 years, except for inflation increases, seriously harming education, social assistance, and healthcare [22].

The shift in the government’s orientation was also evident in healthcare policy. Temer selected as his Minister of Health Ricardo Barros, a legislator with ties to private health insurance corporations, who defended austerity and criticized both the constitutional enshrinement of social security and SUS. Barros advocated the expansion of private health plans and created a commission to develop a proposal for “accessible private health plans,” that is, low-cost, state-subsidized private plans for low-income Brazilians. Accomplishing would have required slacking the requirements of the 1998 Health Insurance Plan Law’s minimum operating criteria for private health insurance plans and consumer rights. After some amendments to the proposal, regulatory measures favorable to private healthcare corporations were adopted.

Changes were also made to key policies covering, inter alia*,* primary care and mental health, which specialists have criticized for conflicting with SUS guidelines or representing setbacks to the previous model of healthcare. The sum of the Temer government’s economic and social austerity measures 2016–2018 has already brought repercussions for several health indicators, as shown below.

### Universal health system and health inequalities: achievements and limits

Since the Constitution of 1988, the recognition of health as a right of citizenship and the struggles for SUS implementation have resulted in important achievements in healthcare access and health status. Moreover, the nationwide expansion of public health programs and health services to new areas and vulnerable social groups has helped to reduce inequalities across regions and among social groups.

There was a massive expansion of health care from 1990 to 2017, comprising both public and private facilities. The most remarkable increases were in basic health services (health centers, health posts, family health units), more than 99% of which are public. This has meant improvements in access to publicly provided primary health care. Private practices and polyclinics have also expanded, most of them contracted by private health insurance plans or paid out-of-pocket by clients. As to hospitals, many municipal facilities were opened, but private units are still predominant, most of them providing services exclusively for SUS or for both SUS and the private sector. Diagnosis and therapy support service units are mainly private as well, generally providing services for the private sector or for both the private sector and SUS [23]. All this shows how the public and the private healthcare organizations and services in Brazil are deeply interconnected.

**Fig. 1:**
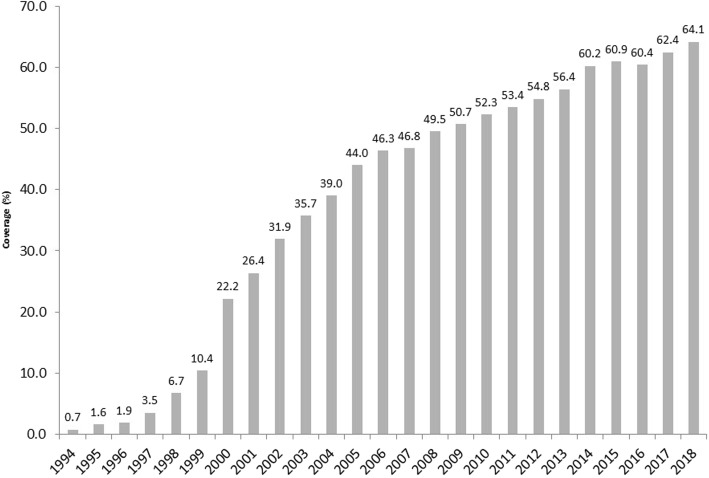
Coverage (%) of Family Health Strategy: Brazil, 1994–2018. Source: Elaborated by the authors. Data from: Basic Health Care Department, Ministry of Health, Brazil (DAB/SAS/MS). From 2002 to 2018. Data available at: http://sage.saude.gov.br/#. Accessed: 07 Set 2019

Expansion of primary healthcare coverage expansion, especially through the Family Health Strategy, has been important nationwide (Fig. 1). This has been more accentuated in economically less-developed regions, particularly among low-income groups [24], with some redistributive effect for federal resources [25].

Many positive outcomes of SUS have been reported, including progressive increases in immunization coverage for a range of diseases and lower rates of preventable hospitalizations [26].

Concerning health status, several studies have noted how SUS has promoted positive health results. These include decreases in overall mortality rates, in infant and child morbidity and mortality, in maternal mortality [27], in mortality due to infectious diseases (especially vaccine-preventable diseases, diarrhea, and respiratory infections) [28], and even in mortality due to some cardiovascular and chronic respiratory diseases [29]. These impressive results can be attributed, in part, to specific programs implemented during the period under study here. For instance, child malnutrition fell sharply; the prevalence of smoking among adults dropped from around 35% in 1989 to 15% in 2012 due to tobacco-control policies; and the incidence of HIV infection has fallen, although recent trends may be worrisome [29].

Similarly, decreases have been reported in inequalities in many health indicators across regions and states. Many of the states that presented the worst mortality indicators in 1990—especially those from the Northeast region—experienced the most significant improvements. For instance, regarding mortality rates for children under five years, the gap between the states with the highest and the lowest levels was almost halved—from a 4.9-fold difference to a 2.5-fold one between 1990 and 2015 [29].

**Fig. 2:**
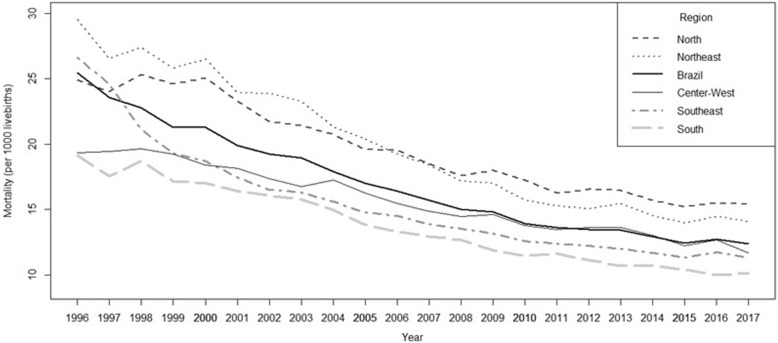
Infant mortality trends: Brazil and its regions, 1996–2017. Source: Elaborated by the authors. Data from: Brazil, Ministry of Health: http://datasus.saude.gov.br/informacoes-de-saude/tabnet/estatisticas-vitais. Accessed 15 Oct 2018

There was an important decline in infant mortality rates across all regions between 1996 and 2015, but most prominently in the Northeast. Then, however, in the wake of the economic recession and the impeachment of Rousseff in 2016, infant mortality rates increased in all regions of the country, except for the highly developed South (Fig. 2).

From 1996 to 2015 infant mortality was statically descendent in Brazil as a whole (β = − 0.65, *p* < 0.001) and in all the five regions, varying from Center-West (β = − 0.41 *p* < 0.001) to Northeast Region (β = − 0.83,*p* < 0.001). In 2016 there was a minor increase (from 12.4 to 12.7 per 1000 live births overall), with the greatest increases occurring in the Northeast and Center-West regions (3.4 and 3.6%, respectively). Again, the sole exception was in the South region, where infant mortality rates continued to fall. Many deaths in 2016 occurred during the post-neonatal period (after the first 28 days of life), with diarrhea as the primary cause [30]. In 2017 the rates tended to remain stable with exception of Center-West, where there was a clear decrease. These oscillations in infant mortality trends may indicate that living standards in the country are falling, particularly among the poor, who have been severely affected by the austerity measures implemented since 2016. Also other sensitive health indicators have shown a recent increase: for example, violence-related mortality in the 15–24 age range [30].

A recent micro-simulation study compared projections of under-five child mortality rates in two different scenarios. The first assumed reductions in the coverage of *Bolsa Família* (Brazil’s conditional cash transfer social welfare program) and the Family Health program due to fiscal austerity; the second scenario hypothesized the maintenance of existing levels of social protection. The authors concluded that the implementation of fiscal austerity measures in Brazil could be held responsible for substantively higher childhood morbidity and mortality [31].

Regarding life expectancy, although a significant decrease occurred during this period, there was considerable variation among geographic regions. In 2013, life expectancy at birth for children born in the richest regions was 76.9 years, as against 71.5 in the least developed regions [32].

In summary, due in part to the implementation of SUS, Brazil witnessed important health advances, which can still be observed across regions and socioeconomic categories. However, the highest rates of illness are still found in the North and Northeast regions, the country’s poorest [33]. Further progress in reducing health inequalities has been obstructed by structural inequities, as well as political decisions that have limited the reach of public funding and promoted the increase of private-sector involvement.

**Fig. 3:**
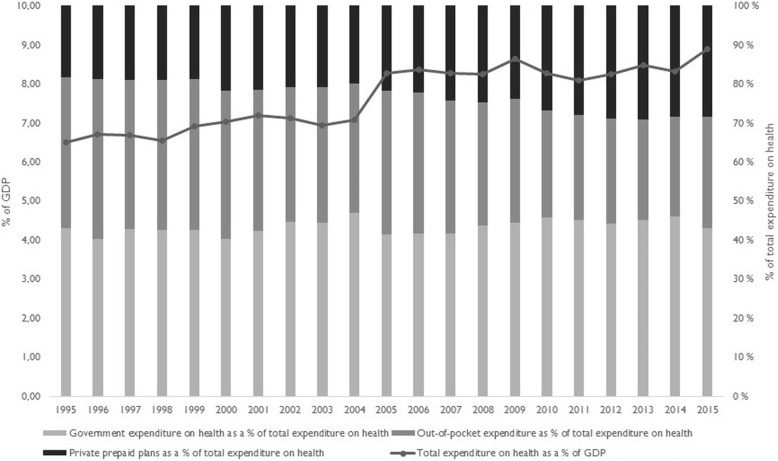
Health expenditures as % of GDP and public–private participation. Brazil, 1995–2015. Source: Elaborated by the authors. Data from World Health Organization. Global Health Observatory. Data Repository. Available at: http://apps.who.int/gho/data/ node.home. Accessed: Oct 2018

As Fig. 3 shows, although total health expenditures as a proportion of GDP increased from 1995 to 2015, private expenditures remained above 50% of total expenditures throughout the period.

**Fig. 4:**
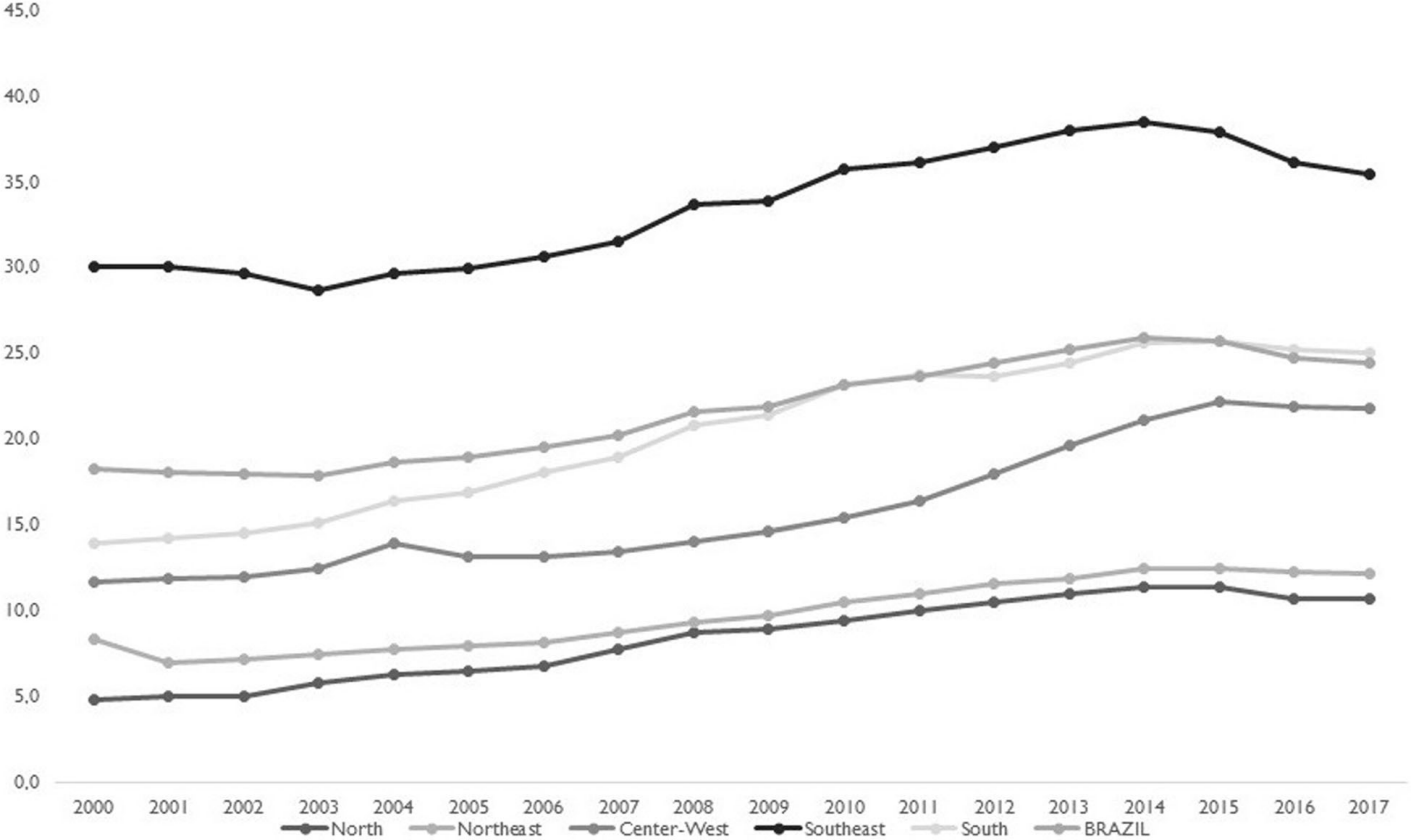
Population coverage (%) of private health plans or insurance. Brazil and its regions, 2000–2017. Source: Elaborated by the authors. Data from Brazil, National Agency of Supplementary Health, ANS Tabnet. http://www.ans.gov.br/anstabnet/#. Accessed 15 Oct 2018

The greatest proportion of expenditure on private healthcare concerns payments for private insurance plans, which increased during this period. By 2017, close to one-fourth of Brazil’s population—over 47 million people—was covered by private plans, although with regional variations, as shown in Fig. 4. With expansion of SUS and the private insurance sector, out-of-pocket expenditures fell, but remained high, particularly for prescription drugs.

## Discussion

The construction of a universal health system in Brazil over the past three decades has been unique in Latin America. The country’s universalist health reform began in the 1980s, as other national health systems were suffering the effects of neoliberal reforms. Democratization created an environment in which political actors dedicated to the defense of health as a citizenship right managed to occupy strategic spaces, from which they influenced policy as well as the 1988 Constitution. In the ensuing decades, under democratic governments, political struggles over a universal health system facilitated the expansion of the public system, with subsequent improvements in health outcomes and some reduction in regional inequalities, when assessed by selected health indicators.

Nevertheless, Brazil still has severe health inequalities [34], due to in part to structural factors, such as the country’s position in the global economy, its own historical particularities, and the characteristics of its systems of social protection and healthcare. However, political variables must also be taken into consideration in explaining the persistence of social inequalities that manifest themselves strongly in the area of health.

In their comparative study of Latin American social policies, Huber and Stephens [35] have shown that democracy was an important factor in explaining the redistributive or non-redistributive nature of social policies. They argue, however, that in the case of Latin America time does matter: longer periods of democratic stability are necessary—estimated at 20–25 years, at a minimum—to identify clearly the effects of social policies on the reduction of inequalities. This occurs, they explain, because democratic stability is a fundamental requirement for new social groups to gain access to power. These groups, through representation or direct participation, are able to influence social policies not merely by expanding them, but by promoting policies aimed at reducing the gaps between rich and poor across various dimensions.

Our examination of the case of the Brazilian health system corroborates Huber and Stephens’ argument. The return to democracy proved fundamental in mobilizing societal actors in defense of the constitutional recognition of health as a right, as well for the construction of an institutional framework for SUS. The period of democratic stability between 1988 and 2015 facilitated the expansion of universalist health policies and services, improvements in health conditions and even some reduction in health inequalities, as has been internationally recognized [34].

This period was also marked by moments of economic crisis, reductions in public spending, and measures aimed at facilitating expansion of the private healthcare market. Still, conflicting agendas and interests notwithstanding, we can note incremental advances in living standards and the reduction of health inequalities. The positive health outcomes registered are also consistent with the findings of a recent observational study which explored the relationships between democratic experience, adult health, and cause-specific mortality in 170 countries, 1980–2016 [36]. Comparing countries with different political regimes, the authors concluded that democracies are more likely than autocracies to lead to health gains for mortality causes requiring healthcare delivery infrastructure, such as cardiovascular diseases and transport injuries.

From 2016, the new political climate surrounding the controversial presidential impeachment, supported by a neoconservative, neoliberal coalition, with democracy under threat, made possible the accelerated adoption of economic austerity and regressive social reforms. In only a short time, it was possible to observe worsening social indicators, such as rates of poverty and extreme poverty, along with stagnation in the reduction of social inequalities that had occurred between 1990 and 2014 [37].

Brazil’s health sector was not immune to the adverse political and economic context. Health indicators—such as infant mortality due to preventable causes, like diarrhea—that had shown continuous improvement since the creation of SUS stagnated or worsened. Although these changes are recent and merit closer study, they would indicate that the advances made possible by SUS have not been entirely sustainable in the face of an adverse economic model. Over the three decades of SUS implementation, on the heels of a situation characterized by deep poverty and inequality, gradual advances were facilitated by the promulgation of the 1988 Constitution and the intense mobilization of the health sector in support of a universalist agenda, putting pressure on democratically elected governments. Lately, however, with political instability and new threats to the social democratic pact of the 1988 Constitution, Brazil has experienced rapid setbacks that have affected the most vulnerable.

Alongside recent developments, it is important to recognize the political struggles over conflicting agendas that occurred throughout the period when SUS was being implemented. These contradictions manifested themselves most strongly in connection with financing, and the relationship between the public system and private sector. Public financing was never sufficient for achieving the goal of a universal system that would reduce social inequalities. The dynamism of the private sector was a pre-SUS legacy, but also a result of international and domestic health insurance companies adopting new business and lobbying strategies to expand their markets and increase profits. In the relationship between the state and health markets, the incentives granted by the former to the latter predominated, abetted by the weakness of regulatory policies.

The political determinants of health inequalities exist on two interrelated levels. The first level concerns the general inequalities of Brazilian society, which, to be modified, would require structural changes in the pattern of development, in turn requiring political consensus on the need to redistribute power and wealth. Such a consensus appears unlikely in the face of the recent rightist consolidation of power: first, after the 2016 impeachment, when Michel Temer assumed the presidency, and then with the 2018 election of the far-right ex-military officer Jair Bolsonaro and an ultraconservative Congress. As of 2019 it seems highly likely that neoliberal policies of economic austerity, combined with continuing uncertainty about the future of Brazilian democracy, will condition the possibilities for social mobilization and resistance to these reforms.

The second level is that of the health sector itself. The political coalitions that influence health policy have changed: the broad political coalition that defended the right to health in the 1980s, which had included academics, health professionals, bureaucrats, social movements and ‘center’ politicians, did not survive. Although the political support to SUS was maintained among health workers, government coalitions involved alliances with conservative sectors and adopted market-oriented reforms detrimental to the public system, and the private health industry became stronger. The adverse post-2016 context exacerbated pre-existing contradictions in the Brazilian health system. The most notable of these was the co-existence of a universal public system with a vigorous and dynamic private sector, which preyed upon SUS by competing with it for state resources and clients, while prioritizing profit. Remedying this situation would require intense social mobilization in defense of the public, universal SUS, and strengthened regulation of the private sector, aimed at containing its growth and subordinating it to the public interest.

In today’s unfavorable global political context, with regressive attacks on social protection in various capitalist countries, it is essential to reflect on the possibilities and limits of political agency for the promotion of social welfare [38]. As Deaton has noted, worldwide improvements in health conditions at certain historical moments have not eliminated the immense gaps between or within rich and poor countries [39].

## Conclusions

A combination of long-term structural and contingent factors, international agendas and interests, as well as domestic political struggles, can explain the advances and obstacles to building a universal system in Brazil, an economically important yet unequal peripheral country. Democracy and political mobilization were essential to implementation of its Unified Health System (SUS) and consequent improvements in health conditions. However, obstacles to structural change persisted, with sustained effects on health inequalities.

Further consolidation of SUS and reduction of health inequalities hinge on the uncertain future of Brazil’s democracy and national development project, on enlarging the political coalition to support a public and universal health system, and on strengthening the state’s ability to regulate the private sector. This analysis of the Brazilian case shows that reducing health inequalities in the face of the dynamics of the global capitalist economy is a major challenge, and one in which politics plays a defining role.

## Data Availability

The datasets analyzed during the current study are available in open access repositories, as quoted and listed in the ‘References’ section or below the Figures.

## Abbreviations

ABRASCO: Brazilian Postgraduate Association in Collective Health
AIDS: Acquired Immune Deficiency Syndrome
CEBES: Brazilian Center for Health Studies
HIV: Human Immunodeficiency Virus
ISI: Import Substitution Industrialization
LGBTIQ+: Lesbian, Gay, Bisexual, Transgender, Intersex and Queer
NHS: National Health Service
PT: Worker’s Party
SUS: Unified Health System
UN: United Nations
